# Prognostic Value of Acute Kidney Injury after Cardiac Surgery according to Kidney Disease: Improving Global Outcomes Definition and Staging (KDIGO) Criteria

**DOI:** 10.1371/journal.pone.0098028

**Published:** 2014-05-14

**Authors:** Maurício N. Machado, Marcelo A. Nakazone, Lilia N. Maia

**Affiliations:** 1 Division of Cardiac Surgery Intensive Care Unit and Coronary Care Unit, Hospital de Base, São José do Rio Preto Medical School, São José do Rio Preto, São Paulo, Brazil; 2 Division of Centro Integrado de Pesquisa (CIP), Hospital de Base, São José do Rio Preto Medical School, São José do Rio Preto, São Paulo, Brazil; San Raffaele Scientific Institute, Italy

## Abstract

**Objectives:**

The definition of acute renal failure has been recently reviewed, and the term acute kidney injury (AKI) was proposed to cover the entire spectrum of the syndrome, ranging from small changes in renal function markers to dialysis needs. This study was aimed to evaluate the incidence, morbidity and mortality associated with AKI (based on KDIGO criteria) in patients after cardiac surgery (coronary artery bypass grafting or cardiac valve surgery) and to determine the value of this feature as a predictor of hospital mortality (30 days).

**Methods:**

From January 2003 to June 2013, a total of 2,804 patients underwent cardiac surgery in our service. Cox proportional hazard models were used to determine the association between the development of AKI and 30-day mortality.

**Results:**

A total of 1,175 (42%) patients met the diagnostic criteria for AKI based on KDIGO classification during the first 7 postoperative days: 978 (35%) patients met the diagnostic criteria for stage 1 while 100 (4%) patients met the diagnostic criteria for stage 2 and 97 (3%) patients met the diagnostic criteria for stage 3. A total of 63 (2%) patients required dialysis treatment. Overall, the 30-day mortality was 7.1% (2.2%) for patients without AKI and 8.2%, 31% and 55% for patients with AKI at stages 1, 2 and 3, respectively. The KDIGO stage 3 patients who did not require dialysis had a mortality rate of 41%, while the mortality of dialysis patients was 62%. The adjusted Cox regression analysis revealed that AKI based on KDIGO criteria (stages 1–3) was an independent predictor of 30-day mortality (P<0.001 for all. Hazard ratio = 3.35, 11.94 and 24.85).

**Conclusion:**

In the population evaluated in the present study, even slight changes in the renal function based on KDIGO criteria were considered as independent predictors of 30-day mortality after cardiac surgery.

## Introduction

Acute kidney injury (AKI) is a complex syndrome that occurs under wide variety of conditions, with manifestations ranging from a small increase in serum creatinine (SCr) to anuric renal failure. The clinical outcomes of this disease range from full recovery to death and might include the development of chronic kidney disease and progression to renal replacement therapy (RRT) [1]. Most studies have diagnosed AKI according to changes in SCr, absolute levels of creatinine, changes in urine output or the need for RRT [2]–[6]. AKI is a common complication in critically ill patients, which generates increased hospital costs [7] and is associated with high mortality as an independent predictor of death [8].

Multinational and multicenter epidemiological studies indicate that sepsis is currently the most common cause of AKI in intensive care, followed by AKI associated with cardiac surgery [9]. Minimal changes in postoperative SCr have been associated with a significant reduction in short and long-term survival [10]. The elevation of SCr might be associated with increased morbidity and mortality, even when these changes do not exceed normal values [11].

Several consensus definitions have been developed to provide uniform criteria for the diagnosis of AKI, facilitating comparisons between studies and the development of quantitative research. In 2004, the “Acute Dialysis Quality Initiative (ADQI)” proposed guidelines, called RIFLE criteria (Risk, Injury, Failure, Loss and End-stage Kidney Disease) [12], subsequently modified by the “Acute Kidney Injury Network” (AKIN, which included the ADQI) [13]–[15]. More recently, the AKI study group, “Kidney Disease: Improving Global Outcomes (KDIGO)”, has suggested a modified definition, harmonizing the differences between the RIFLE and AKIN definitions [1]. These definitions were independently validated in multiple studies and are now widely accepted [2], [16].

The aim of the present study was to apply the AKI criteria, based on KDIGO classification, in a population of patients undergoing cardiac surgery [coronary artery bypass grafting (CABG) or cardiac valve surgery (CVS)] to evaluate the impact of this feature as a predictor of 30-day mortality.

## Materials and Methods

### Patient Selection

**Figure 1:**
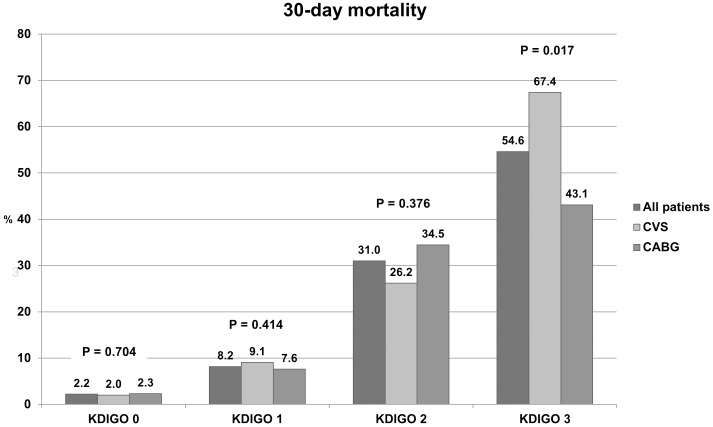
30-day mortality of cardiac surgery patients based on KDIGO classification.

We conducted a single-center study. We retrospectively evaluated patients from the Cardiac Surgery Intensive Care Unit in a Brazilian Medical School facility. The demographics, type of surgery, laboratory data and preoperative, perioperative, and postoperative information were retrieved from a prospectively collected database of 2,878 patients older than 18 years and undergoing isolated CABG (1,786) or CVS (1,092) from January 2003 to June 2013. After applying the exclusion criteria (51 patients with incomplete data and 23 patients with end-stage kidney disease), a total of 2,804 patients were suitable for analysis: 1,738 (62%) patients underwent CABG, and 1,066 (38%) patients underwent CVS (Figure 1).

This study was conducted in accordance with the Declaration of Helsinki and approved through the Local Human Research Ethics Committee of São José do Rio Preto Medical School (protocol 5974/2008). The need for individual informed consent was waived, as this study was a retrospective analysis of prospectively collected data for routine care, and there was no breach of privacy or anonymity. ClinicalTrials.gov register – NCT00777465.

### Serum Creatinine Measurement

The Jaffe colorimetric method (ADVIA 1650, Bayer, Germany) was used to measure the SCr concentration. The reference value for adults is 0.6 to 1.3 mg/dL for men and 0.6 to 1.0 mg/dL for women.

### Diagnosis and Staging of AKI (KDIGO) [1]

AKI was defined as any of the following:

Increase in SCr≥0.3 mg/dL (≥26.5 µmol/L) within 48 hours; increase in SCr≥1.5 times baseline, which is known or presumed to have occurred within the prior 7 days;

AKI was also staged for severity according to the following criteria:

Stage 1: Increase in SCr≥0.3 mg/dL (≥26.5 µmol/L) or 1.5 to 1.9 times baseline

Stage 2: 2.0 to 2.9 times baseline SCr

Stage 3: 3.0 or more times baseline; increase in SCr≥4.0 mg/dL; or initiation of renal replacement therapy

### Data Analysis

Serum creatinine was measured daily in the postoperative period, according to the intensive care routine, and every 48 hours on the ward. These values were recorded until the 7^th^ day after surgery, and the patients followed during the first 30 days of surgery. The diagnosis of AKI was based on the SCr levels recorded during the first seven days after surgery (compared with the preoperative measurement on admission to the hospital) as well as the need for RRT, and the patients were classified as no AKI (KDIGO 0) or AKI (KDIGO 1–3). Due to the lack of data on urine output, only SCr was used to determine the AKI categories. The glomerular filtration rate (eGFR) was estimated using the Cockcroft-Gault equation [17], and consulting nephrologists in charge of the patient’s care (available 24/7) established the need for RRT. The risk of postoperative death was estimated using EuroSCORE [18], [19], in the absence of a specific tool for our population.

### Statistical Analysis

The data were analyzed using the IBM SPSS Statistical Package v.20 (IBM Corporation, Armonk, NY). The variables are presented as absolute numbers and percentages and median and interquartile ranges (25^th^ and 75^th^ percentile) when applicable. Due to the lack of Gaussian distribution, continuous variables were compared using the nonparametric Mann-Whitney or Kruskal-Wallis tests. Chi-square or Fisher’s exact tests were used to compare categorical variables.

Univariate and multivariable Cox proportional hazards models (stepwise backward elimination method) were used to determine the association between AKI and mortality at 30 days. The model was adjusted for age (years), gender (reference – male gender), type of surgery (reference – CVS), body mass index (kg/m^2^), diabetes mellitus (reference – non-diabetic), left ventricular function (reference – preserved left ventricular ejection fraction), cardiopulmonary bypass (CPB) times (min) and AKI (reference – KDIGO 0). The adjusted Hazard Ratio (HR) and 95% confidence intervals (95% CI) were calculated for the predictors. Cumulative survival graphics were constructed to demonstrate the AKI impact as a predictor of 30-day mortality. P values<0.05 were considered statistically significant (two-tailed).

## Results

The demographic data and kidney function for each patient are described in Table 1. The age of the patients increased between groups as well as the percentage of patients undergoing repeat surgery and intensive care length of stay. Patients with AKI stages 1 to 3 exhibited higher EuroSCORE scores, but there was no difference in the number of patients classified as low, intermediate or high risk between the groups (P = 0.118). Thirty-six percent of the patients had preoperative chronic kidney disease (33% with an eGFR between 30–59 mL/min and 3% with an eGFR≤29 mL/min).

**Table 1:** Baseline characteristics and renal function of 2,804 patients underwent cardiac surgery.

	All patients	KDIGO 0	KDIGO 1	KDIGO 2	KDIGO 3	
	n = 2,804 (100%)	n = 1,629 (58%)	n = 978 (35%)	n = 100 (4%)	n = 97 (3%)	
	Median (IQR)or n (%)	Median (IQR)or n (%)	Median (IQR)or n (%)	Median (IQR)or n (%)	Median (IQR)or n (%)	P Valuefor all
Coronary artery bypass grafting	1,738 (62)	1,037 (64)	592 (61)	58 (58)	51 (53)	0.680
Cardiac valve surgery	1,066 (38)	592 (36)	386 (39)	42 (42)	46 (47)	0.680
Multiple CVS*	385 (36)	181 (31)	163 (42)	18 (43)	23 (50)	<0.001
CVS during active IE*	81 (7.6)	40 (6.8)	27 (7.0)	2 (4.8)	12 (26)	<0.001
Age (years)	59 (49–66)	57 (47–65)	60 (52–68)	63 (55–70)	63 (56–70)	<0.001
Male gender	1710 (61)	963 (59)	628 (64)	55 (55)	64 (66)	0.027
Weight (kg)	70 (61–80)	70 (61–80)	70 (61–80)	72 (63–83)	71 (60–85)	0.218
Height (m)	1.65 (1.57–1.70)	1.65 (1.57–1.70)	1.65 (1.58–1.70)	1.64 (1.55–1.68)	1.67 (1.60–1.73)	0.112
Body mass index (kg/m^2^)	26 (23–29)	26 (23–29)	26 (23–29)	28 (24–32)	26 (23–30)	0.004
Diabetes Mellitus	634 (23)	338 (21)	242 (25)	34 (34)	20 (21)	0.004
LV dysfunction (Moderate/severe)	568 (20)	304 (19)	214 (22)	24 (24)	26 (27)	0.055
Intra-aortic balloon pump**	146 (8.4)	66 (6.4)	58 (10)	10 (17)	12 (24)	<0.001
Preoperative SCr (mg/dL)	1.10 (0.90–1.30)	1.10 (0.90–1.30)	1.10 (0.90–1.30)	1.00 (0.80–1.28)	1.20 (1.00–1.60)	<0.001
Preoperative eGFR (mL/min)	68 (53–87)	69 (54–87)	67 (51–88)	69 (58–97)	59 (39–79)	<0.001
≥90 mL/min	647 (23)	375 (23)	224 (23)	30 (30)	18 (19)	
60–89 mL/min	1,163 (41)	721 (44)	371 (38)	41 (41)	30 (31)	<0.001
30–59 mL/min	926 (33)	506 (31)	355 (36)	29 (29)	36 (37)	
≤29 mL/min	68 (3.0)	27 (1.7)	28 (2.9)	0 (0.0)	13 (13)	
Immediate PO SCr (mg/dL)	1.10 (0.90–1.40)	1.00 (0.90–1.20)	1.30 (1.10–1.50)	1.20 (1.00–1.50)	1.30 (1.10–1.90)	<0.001
Repeat surgery	339 (12)	146 (9.0)	143 (15)	19 (19)	31 (32)	<0.001
Additive EuroScore	3 (2–5)	3 (1–5)	4 (2–6)	4 (2–6)	6 (4–9)	<0.001
Low risk (<3)	1,064 (38)	708 (43)	316 (32)	28 (28)	12 (12)	
Intermediate risk (3 to 5)	1,133 (40)	664 (41)	406 (42)	35 (35)	28 (29)	0.118
High risk (>5)	607 (22)	257 (16)	256 (26)	37 (37)	57 (59)	
ICU length of stay (days)	2 (2–5)	2 (2–4)	3 (2–6)	4 (2–12)	7 (3–17)	<0.001

IQR – interquartile ranges; CVS – cardiac valve surgery; IE – infective endocarditis; LV – left ventricular; SCr – serum creatinine; eGFR – estimated glomerular filtration rate; PO – postoperative; ICU – intensive care unit.

*The percentages are related to cardiac valve surgery patients;

**The percentages are related to coronary artery bypass grafting patients.

The prevalence of AKI was 42% distributed in 35% of patients with AKI stage 1, 4% of patients with AKI stage 2 and 3% of patients with AKI stage 3. A total of 63 (2%) patients required dialysis treatment, representing sixty-five percent of stage 3 patients. The development of AKI stage 3 was most frequently observed in patients with impaired eGFR (≤29 mL/min) (1.7% vs. 13%) and CPB times greater than 120 minutes (13% vs. 47%; Table 2).

**Table 2:** Cardiopulmonary bypass times, complications and mortality of patients underwent cardiac surgery.

	All patients	KDIGO 0	KDIGO 1	KDIGO 2	KDIGO 3	
	n = 2,804 (100%)	n = 1,629 (58%)	n = 978 (35%)	n = 100 (4%)	n = 97 (3%)	
	Median (IQR)or n (%)	Median (IQR)or n (%)	Median (IQR)or n (%)	Median (IQR)or n (%)	Median (IQR)or n (%)	P Value for all
On-pump CABG	2,381 (85)	1,311 (80)	891 (91)	91 (91)	88 (91)	<0.001
Cardiopulmonary bypass time	94 (78–113)	90 (76–109)	97 (80–114)	103 (85–127)	121 (90–155)	<0.001
<90 min	993 (41)	619 (38)	330 (34)	26 (26)	18 (19)	
90 to 120 min	932 (39)	493 (30)	380 (39)	35 (35)	24 (25)	0.001
>120 min	465 (20)	206 (13)	183 (19)	30 (30)	46 (47)	
Off-pump CABG	414 (15)	311 (19)	85 (9.0)	9 (9.0)	9 (9.0)	<0.001
Acute atrial fibrillation	255 (9.1)	98 (6.0)	108 (11)	21 (21)	28 (29)	<0.001
Tracheal re-intubation up to 7th day	183 (6.5)	47 (2.9)	81 (8.3)	21 (21)	34 (35)	<0.001
Mechanical ventilation >24 hours	291 (12)	67 (4.7)	123 (15)	34 (40)	67 (77)	<0.001
30-day mortality	200 (7.1)	36 (2.2)	80 (8.2)	31 (31)	53 (55)	<0.001

IQR – interquartile ranges.

Patients with no postoperative AKI had a 2.2% mortality rate (36 deaths out of 1,629 patients), while patients with postoperative AKI had a 14% overall mortality rate (164 deaths out of 1,175 patients). Any degree of AKI was associated with a significant increase in the overall 30-day mortality (Table 2). The KDIGO stage 3 patients who did not require dialysis had a mortality rate of 41%, while the mortality of dialysis patients was 62%.

The subgroup analysis (CABG and CVS) showed similar results with worse mortality in patients undergoing CVS who developed AKI stage 3 (CABG – 43.1% vs. CVS – 67.4%, P = 0.017; Figure 1). Among CVS patients with AKI, the proportion of individuals subjected to repeat and multiple valve surgeries and surgery during active endocarditis increased progressively, with worsening renal function (Table 1).

### Multivariable Cox Proportional Hazards Model

**Figure 2:**
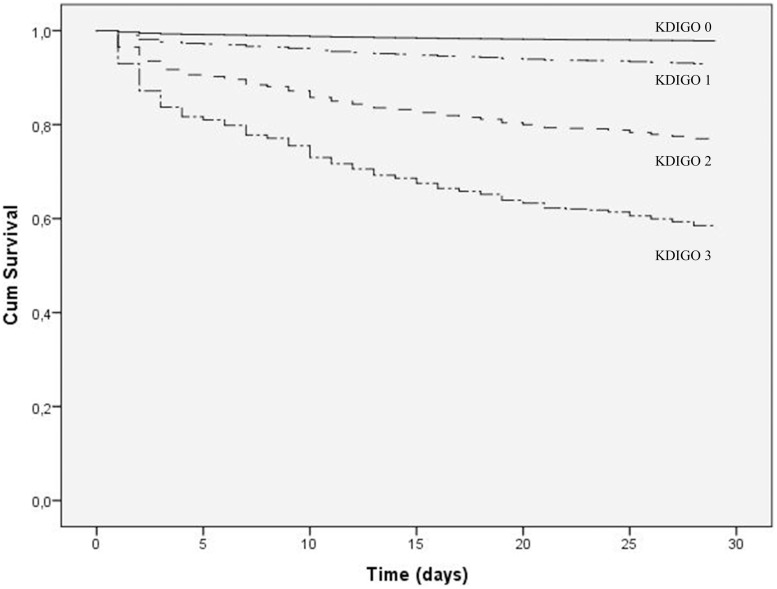
Overall survival curves when the patients were divided into four groups according to the KDIGO criteria.

The multivariable Cox proportional hazards analysis showed that age (years), female gender, CPB time (min) and AKI (KDIGO stages 1–3) were independent predictors of 30-day mortality. The HR (and 95% CI) for overall mortality according to the AKI stages was (reference - KDIGO 0) stage 1, 3.35 (2.19 to 5.12); stage 2, 11.94 (7.05 to 20.20); and stage 3, 24.48 (15.05 to 39.81). Female patients showed a 63% increased risk of death at 30 days (Table 3; Figure 2). The subgroup analysis (CABG and CVS) showed similar results, except for the increased risk of death observed in female CABG patients (data not shown). When we analyzed patients based on baseline eGFR (above and below 60 mL/min), AKI staging remained an independent predictor of death, with robust results in patients with previously preserved renal function [HR – 3.08, 17.51 and 48.86 for stages 1, 2 and 3, respectively (eGFR≥60 mL/min) and HR – 3.47, 11.88 and 16.75 for stages 1, 2 and 3, respectively (eGFR<60 mL/min) – P<0.001 for all].

**Table 3:** All patients – Multivariate analysis by Cox proportional hazards models – Hazard Ratio and 95% confidence intervals for predictors of 30-day mortality after cardiac surgery.

	HR (95% CI)	P Value
Age (years)	1.04 (1.03–1.06)	<0.001
Female gender	1.63 (1.22–2.17)	0.001
Cardiopulmonary bypass times (min)	1.01 (1.00–1.01)	<0.001
KDIGO 0	Reference	
KDIGO 1	3.35 (2.19–5.12)	<0.001
KDIGO 2	11.94 (7.05–20.20)	<0.001
KDIGO 3	24.48 (15.05–39.81)	<0.001

HR – Hazard Ratio; CI – confidence interval.

## Discussion

In the present study, we evaluated patients with AKI after cardiac surgery (CABG or CVS) to evaluate this feature as a predictor of 30-day mortality. To the best of our knowledge, this study is the largest cohort of patients with AKI after cardiac surgery assessed using KDIGO classifications and showing the impact of AKI severity (stages 1–3) as a predictor of hospital mortality. Using the Multivariable Cox Regression Model, we clearly demonstrated that the development of AKI (according to KDIGO criteria) after cardiac surgery is a strong predictor of 30-day mortality as well as age, female gender and CPB times. In our study, a wide variation was observed in patient mortality, as patients who developed stage 3 AKI (97 patients - 3%) had a hospital mortality rate of 55% and a risk of death 24.5 times greater than that of patients with no AKI.

Robert et al. [20] evaluated more than 25,000 patients undergoing cardiac surgery and observed that 3.6% of patients developed AKI stage 3 (AKIN criteria). The univariate analysis revealed a hospital mortality rate of 36.8%, with an odds ratio of 43.8. De Santo et al. [21] evaluated 1,424 patients undergoing cardiac valve surgery and observed a 43.8% hospital mortality rate in patients classified as RIFLE–*Failure*, with an odds ratio of 30.

Even minimal changes in postoperative SCr were associated with a significant reduction in short and long-term survival [10]. The SCr elevation might be associated with increased morbidity and mortality, even when the change did not exceed normal values [10], [11]. After cardiac surgery, AKI might occur in up to 48% of patients [22] and up to 9.6% of patients require RRT, particularly those with preoperative renal dysfunction [23]. In our sample, 2% of patients (65% of those classified as KDIGO stage 3) required RRT, in the first 7 postoperative days. Epidemiological studies have reported an RRT requirement of approximately 2.6% to 4.9% [24], [25]. Our lower incidence of RRT was associated with the fact that the need for dialysis treatment was evaluated only in the first seven days after surgery but the similarity of other studies suggests that although each center has different patient populations and criteria for indicating RRT, the average incidence of severe AKI requiring RRT is approximately 4% [26]. In our study, the mortality of KDIGO stage 3 patients needing RRT peaked at 62%, in contrast with the mortality of patients without postoperative AKI (2.2%). Although the mortality of patients treated with RRT after cardiac surgery declined [2], in most studies, this factor remained greater than 40% [5], [16], [27].

We also found that age, female gender and CPB times are predictors of 30-day mortality after cardiac surgery. Age and female gender are traditional predictors of early and late mortality after cardiac surgery and are present in most contemporary operative risk scores [28], [29]. Many studies have found higher mortality rates after cardiac surgery in female gender [30]–[32] but not all [33]–[35]. The higher mortality could be explained by differences in baseline characteristics such as older age, higher body mass index, more cardiovascular risk factors and comorbidities. Considering these possible confounders, we found that female gender was an independent risk factor for 30-day mortality after cardiac surgery. Cardiopulmonary bypass times were also implied to increase mortality after cardiac surgery [36]. CPB is associated with significant hemodynamic changes, and the maintenance of cardiovascular stability during CPB requires the interplay between the function of the CPB machine and patient factors [37]. Thus, any decrease in renal perfusion during CPB, depending on its magnitude and duration, can lead to significant cellular injury [37].

Currently, only three studies have used the KDIGO classification to evaluate patients after cardiac surgery. In the first study, Ho et al. [38] evaluated the change in SCr (greater or less than 10%) during the first 6 hours after surgery in 350 patients undergoing CABG or CVS. The results showed that 14% of patients developed AKI according to the KDIGO criteria, with greater than 10% variation in SCr immediately after surgery, strongly associated with subsequent AKI after cardiac surgery. In the second study, Sampaio et al. [39] evaluated the incidence and risk factors for AKI in 321 patients after cardiac surgery according to RIFLE, AKIN and KDIGO criteria. The incidence of AKI ranged from 15–51%, and the adjusted Cox regression analysis revealed that only cases diagnosed using the KDIGO criteria remained associated with the composite endpoint of death, the requirement for RRT and prolonged hospitalization. In the last study, Bastin et al. [2] retrospectively evaluated 1,881 patients undergoing cardiac surgery, comparing RIFLE, AKIN and KDIGO classification criteria. The area under the receiver operating characteristic curve for hospital mortality was significantly higher using the AKIN classification compared with the RIFLE criteria. The incidence and outcome of AKI according to AKIN and KDIGO criteria were identical.

AKI after cardiac surgery occurs secondary to renal ischemia, resulting from heart failure, prolonged hypotension or cardiovascular collapse, interruption of renal circulation, vasopressors and “post-pump syndrome” [40]. AKI might also result from atheroembolic renal insult, hemoglobinuria or myoglobinuria, age, hyperbilirubinemia, sepsis, angiotensin converting enzyme inhibitors, angiotensin receptor blockers, and the use of anti-inflammatory non-steroidal or radio-contrast dye immediately prior to surgery [40]. However, the most predictable risk factor for AKI is pre-existing chronic kidney disease [41]. The lack of a widely used classification for AKI in different populations compromises the understanding of the incidence, evolution and effectiveness of therapeutic interventions.

## Strength and Study Limitations

This study is a retrospective analysis of prospectively collected single-center data. Thus, the study design did not facilitate the characterization of potential causes of postoperative AKI, such as hemodynamic, electrolyte and acid-basic disturbances and the use of nephrotoxic or vasoactive drugs. The interpretation and comparison of the results obtained in the present study with those of studies based on different AKI classifications might be impaired.

## Conclusion

In the population evaluated in the present study, even slight changes in renal function based on KDIGO criteria were considered as independent predictors of 30-day mortality after cardiac surgery (CABG or CVS). Age, female gender and CPB times were also independent mortality predictors.
